# Mangosteen Extract Shows a Potent Insulin Sensitizing Effect in Obese Female Patients: A Prospective Randomized Controlled Pilot Study

**DOI:** 10.3390/nu10050586

**Published:** 2018-05-09

**Authors:** Mikiko Watanabe, Elena Gangitano, Davide Francomano, Eliana Addessi, Raffaella Toscano, Daniela Costantini, Dario Tuccinardi, Stefania Mariani, Sabrina Basciani, Giovanni Spera, Lucio Gnessi, Carla Lubrano

**Affiliations:** 1 Department of Experimental Medicine, Section of Medical Pathophysiology, Food Science and Endocrinology, Sapienza University of Rome, 00161 Rome, Italy; Ele.gangitano@hotmail.it (E.G.); Davide.francomano@yahoo.it (D.F.); Eliana.addessi@gmail.com (E.A.); Raffaella.toscano@uniroma1.it (R.T.); D.costantinidc@gmail.com (D.C.); S.mariani@uniroma1.it (S.M.); Sabrinabasciani@yahoo.it (S.B.); Giannispera@yahoo.com (G.S.); Lucio.gnessi@uniroma1.it (L.G.); Carla.lubrano@uniroma1.it (C.L.); 2 Department of Endocrinology and Diabetes, University Campus Bio-Medico of Rome, 00128 Rome, Italy; d.tuccinardi@unicampus.it

**Keywords:** *Garcinia mangostana*, inflammation, insulin resistance, metabolic syndrome, diabetes, xanthones, mangostin, phytotherapy, dietary supplements

## Abstract

There is a widely acknowledged association between insulin resistance and obesity/type 2 diabetes (T2DM), and insulin sensitizing treatments have proved effective in preventing diabetes and inducing weight loss. Obesity and T2DM are also associated with increased inflammation. Mangosteen is a tropical tree, whose fruits—known for their antioxidant properties—have been recently suggested having a possible further role in the treatment of obesity and T2DM. The objective of this pilot study has been to evaluate safety and efficacy of treatment with mangosteen extract on insulin resistance, weight management, and inflammatory status in obese female patients with insulin resistance. Twenty-two patients were randomized 1:1 to behavioral therapy alone or behavioral therapy and mangosteen and 20 completed the 26-week study. The mangosteen group reported a significant improvement in insulin sensitivity (homeostatic model assessment-insulin resistance, HOMA-IR −53.22% vs. −15.23%, *p* = 0.004), and no side effect attributable to treatment was reported. Given the positive preliminary results we report and the excellent safety profile, we suggest a possible supplementary role of mangosteen extracts in the treatment of obesity, insulin resistance, and inflammation.

## 1. Introduction

In recent years, industrialized countries have witnessed a rapid and progressive increase in the prevalence of obesity, both for improved economic conditions and the spread of a sedentary lifestyle. According to the World Health Organization, 39% of adults were overweight, and 13% were obese worldwide in 2016 [1]. Obesity is a chronic disease and is one of the major risk factors for the development of type 2 diabetes (T2DM) and its comorbidities. There is a widely acknowledged association between insulin resistance and obesity/type 2 Diabetes (T2DM) [2], and insulin sensitizing treatments have proved effective in preventing diabetes and inducing weight loss [3,4]. First-line clinical intervention for obesity and T2DM is lifestyle change, but this is insufficient in many patients, and so drug therapy is often needed. Obesity and T2DM are also associated with increased inflammation, with elevations of serum C reactive protein (CRP), plasma fibrinogen, and other acute-phase proteins [5].

*Garcinia mangostana* Linn., also known as mangosteen, is an evergreen tree native to Southeast Asia, whose fruits have been used in traditional medicine to treat several conditions for centuries. The main phytochemicals present in mangosteen are alpha and gamma mangostins, isoprenylated xanthones, a class of secondary metabolites widely known for their antioxidant properties [6], but recent evidence has suggested a possible further role in the treatment of obesity and T2DM. Preclinical studies show in fact glucose lowering properties possibly through an alpha glucosidase activity and pancreatic beta cells hyperplasia in mangosteen treated animals [7,8,9]. Moreover, in vitro evidence suggests that alpha-mangostin is a potent inhibitor of pancreatic lipase, similarly to commercially available anti-obesity drug orlistat [10], and is able to induce apoptosis and lipolysis in preadipocytes through inhibition of fatty acid synthase, potentially inhibiting fat accumulation in vivo [11]. Mangosteen was also reported to reduce inflammation through several pathways such as inhibition of nitric oxide (NO) and prostaglandin E2 (PGE2) release and moderate suppression of pro-inflammatory cytokines (TNF-alpha, IL-6, and IL-1beta) production [12,13,14]. Moreover, animal research conducted on diet induced obesity (DIO) mice treated with alpha-mangostins report weight loss, attenuated hepatic steatosis, decreased serum glucose, and improved lipid profile through sirtuin 1-AMP-activated protein kinase and peroxisome proliferator-activated receptor (PPAR) gamma pathways [15,16]. Pilot studies lasting 4 to 16 weeks conducted on human subjects and assessing the effect of up to 800 mg of mangosteen extracts point in the same direction as preclinical ones, with reported significant improvements in inflammatory markers, weight loss, and waist circumference reduction, and an excellent safety and tolerability profile [17,18,19,20].

To date, no study has been conducted to primarily assess the effect of mangosteen on insulin resistance. The objective of this pilot study has been to evaluate safety and efficacy of treatment with mangosteen extract on insulin resistance, weight management, and inflammatory status in obese female patients with insulin resistance. We report promising results, with a potent insulin reduction.

## 2. Materials and Methods

### 2.1. Patients

Patients were recruited among subjects referring to the High Specialization Center for the Care of Obesity (CASCO) at the Department of Experimental Medicine, Sapienza University of Rome. Inclusion criteria were: female gender, age between 18 and 65 years; obesity (Body Mass Index BMI ≥ 30 kg/m^2^ with body weight less than 135 kg); insulin resistance (HOMA-IR ≥ 2.5); no acute medical conditions requiring hospitalization in the preceding six months. The weight limit was due to the dual-energy X-ray absorptiometry (DXA) scan weight limit. Exclusion criteria were: any medical condition that could preclude patient safety according to the opinion of the physician; diabetes; history of cardiovascular disease; use of medications potentially affecting study outcomes such as weight loss drugs (including supplements), anti-inflammatory (NSAIDs and steroids), insulin sensitizing (metformin), and lipid lowering medications; unstable body weight within the previous three months (≥3 kg change); pregnancy; and absence of informed consent. All participants were asked to sign a written informed consent before the beginning of the trial. The study protocol was conducted according to the principles of the Declaration of Helsinki and was approved by the Ethics Committee of Sapienza University of Rome (ClinicalTrials.gov Identifier: NCT02823561).

### 2.2. Study Protocol

We conducted a 26-week prospective randomized, controlled, parallel group study. Subjects were randomly assigned to two different arms of treatment: standard hypocaloric diet and physical activity or standard hypocaloric diet, physical activity, and treatment with mangosteen supplement 400 mg once daily (OD).

### 2.3. Lifestyle Intervention

A hypocaloric diet was prescribed to all subjects at baseline. 300 kcal/day were subtracted from individual estimated total energy expenditure based on the Harris–Benedict equation [21]. The daily dietary intake included approximately 45–50% of calories from carbohydrate, up to 30% of calories from fat (<10% saturated fat) and 20–25% of calories from protein. Subjects were instructed to have moderate-intensity physical activity (e.g., 30 min walking every day) during the study. Further suggestions were tailored to the patient habits and abilities and included activities falling in the 3–6 METs range that defines moderate intensity level [22]. Patients met individually with a dietician once a month to assess compliance to prescribed diet through a 24 h dietary recall. Physical activity was self-reported and adherence was subjectively evaluated by the dietician at every visit.

### 2.4. Outcome Measures

#### 2.4.1. Anthropometric Measurements and Vital Parameters

Body weight and height were obtained between 8 and 10 AM in fasting subjects wearing light clothing and no shoes with an empty bladder at baseline and at every monthly follow up visit. The same calibrated scale and stadiometer were used for all patients. Waist circumference was measured in the same instance at the midpoint between the lower rib margin and the iliac crest, the patients had their waist uncovered and were asked to stand with their feet close together and their weight equally distributed on each leg. Systolic and diastolic blood pressure were measured using a calibrated device under standardized conditions, on the same arm, at baseline and at every monthly follow up visit. Heart rate was measured over 1 min at the time of the measurement of blood pressure.

#### 2.4.2. Laboratory Assessments

Blood samples were collected from fasting patients by venipuncture between 8 and 9 a.m. at baseline and at the end of the 26 week study. Samples were then transferred to the local laboratory and handled according to the local standards of practice. Insulin, glucose, glycosilated hemoglobin A1C (HbA1C), inflammatory markers (high sensitivity CRP (hsCRP), fibrinogen), lipid profile (total cholesterol, high-density lipoproteins (HDL) and low-density lipoproteins (LDL) cholesterol, triglycerides), and routine laboratory tests were measured. Homeostasis model assessment of insulin resistance (HOMA-IR) was calculated from fasting plasma insulin and glucose levels using the following formula: insulin × glucose/405 (mIU/L × mg/dL) [23].

#### 2.4.3. Body Composition

Body composition was measured by dual X-ray absorptiometry (DXA) (QDR Discovery Acclaim, Hologic Inc., Waltham, MA, USA) in fasting patients wearing light clothing and no shoes at baseline and at the end of the 26-week study.

### 2.5. Product Description

The active ingredient, in a capsule formulation, was *Garcinia mangostana* 400 mg, titrated to 40% in alpha and gamma mangostins (Osebo, Sanamedica Group srl, Rome, Italy) (Table 1). The Patients were instructed to take one capsule at lunch every day. To assess compliance, patients were asked to bring back any left capsules to the monthly visit and to report if and when treatment had not been taken at lunch as prescribed. Patients in the control group did not receive placebo treatment and only benefited from lifestyle intervention.

### 2.6. Statistical Analysis

Expecting a baseline mean HOMA-IR of 4 ± 1.5 in obese insulin resistant patients, a sample size of nine patients per group was calculated to detect a 40% HOMA-IR decrease in the treatment group compared to control with an α of 0.05 and a (1-β) of 80%. With a foreseen 20–30% dropout rate, 22 patients were enrolled and randomized 1:1 in the two treatment groups. Statistical tests were performed using GraphPad Prism Version 5.00 for Windows, GraphPad Software, San Diego California USA and SPSS Statistics for Windows, Version 20.0, Armonk, NY, USA: IBM Corp. All results are expressed as mean ± standard deviation (SD). Differences obtained in the two groups after 26 weeks of treatment were evaluated by two-way ANOVA variance analysis. When control for covariates (BMI or weight loss) was needed, two-way ANCOVA variance analysis was performed. Differences over time within a group were assessed with a paired sample two-tailed Student’s *t*-test. Normality was assessed with the Shapiro–Wilk test. Differences were considered statistically significant when *p* < 0.05.

## 3. Results

### 3.1. Population

After a clinical assessment and evaluation of inclusion and exclusion criteria, 22 obese female patients (BMI ≥ 30 Kg/m^2^), aged between 18 and 65, were enrolled and randomized 1:1 to mangosteen and lifestyle intervention (*n* = 11), the treatment group, or lifestyle intervention only, the control group (*n* = 11), between November and December 2015 at the Centre of High Specialization for the Cure of Obesity (CASCO), Sapienza University of Rome, Italy. We had initially planned a mixed gender trial, but the enrolled subjects were significantly more female than male, and we therefore opted for a more homogeneous sample by excluding male patients as the field of study is new and the sample size relatively small, although no gender difference has been reported so far in regard to mangosteen treatment. Two subjects (one belonging to the mangosteen group and one to the control group) did not complete the study due to personal reasons and were therefore excluded from the analysis. The groups were not significantly different at baseline in regard to age, BMI, body composition, fasting glucose, and insulin levels and hsCRP (Table 2).

### 3.2. Glucose Metabolism

**Figure 1:**
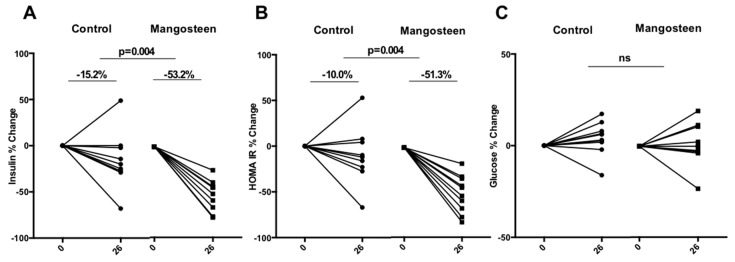
Glucose metabolism markers. (**A**) Insulin levels decreased significantly in the treatment group compared to control at 26 weeks; (**B**) HOMA IR % change went in the same direction in favor of the mangosteen group that showed a frank improvement in insulin resistance; (**C**) glucose levels did not significantly change in any of the studied arms.

Insulin levels at 26 weeks decreased significantly in the treatment group compared to control (−53.2% vs. −15.2%, *p* = 0.004) (Figure 1A). HOMA IR% change went in the same direction with a reduction of −51.3% vs. −10% (*p* = 0.004 Figure 1B) in favor of the mangosteen group that showed a frank improvement in insulin resistance. These results remained significant after correction for BMI change or weight loss over time. Glucose levels did not significantly change in any of the studied arms (Figure 1C).

### 3.3. Anthropometric Parameters

**Figure 2:**
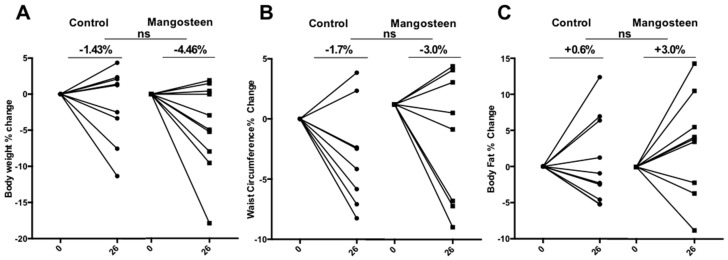
Anthropometric parameters: (**A**) the mangosteen arm experienced weight loss (−4.5 ± 6.2%, *p* = 0.048) that the control failed to do, however groupwise comparison was not significant; (**B**) no statistically significant difference was seen regarding waist circumference in any of the groups; (**C**) no statistically significant difference was seen regarding body fat percentage in any of the groups.

The mangosteen arm experienced a weight loss (−4.5 ± 6.2%, −4.07 ± 6.01 kg; *p* = 0.048) that the control failed to show (−1.42 ± 4.9%, −0.88 ± 4.51 kg; *p* = 0.420) (Figure 2A), but groupwise comparison was not significant. No statistically significant difference was seen regarding waist circumference and body composition in any of the groups, with the control group showing a reduction in fat and lean mass of −0.54 ± 4.04 kg and −2.16 ± 5.51, respectively; and the mangosteen group showing a reduction of −1.18 ± 5.48 and −1.91 ± 7.19 of the same parameters (Figure 2B,C).

### 3.4. Inflammation Markers

**Figure 3:**
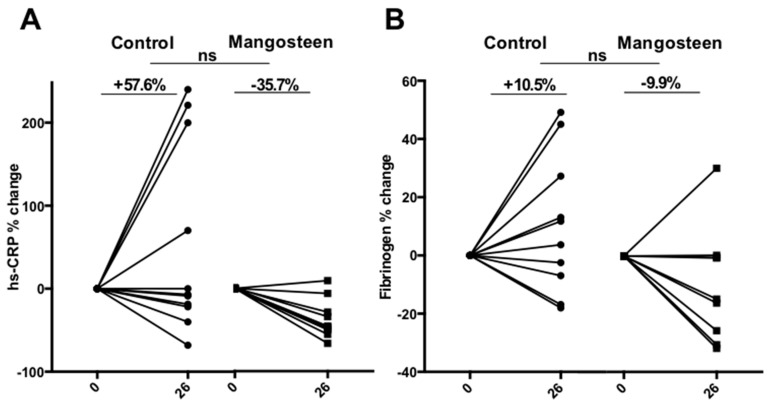
Inflammation markers: (**A**) HsCRP was significantly reduced in the mangosteen group, with a mean decrease of 0.41 ± 0.34 mg/L (*p* = 0.004, −35.7 ± 22.51%). Comparison with the control group failed to show any significant groupwise difference (*p* = 0.13); (**B**) Fibrinogen levels had a trend decrease in the mangosteen group (−57 ± 93 mg/L, −9.9 ± 19.0%, *p* = 0.100) but failed to be significantly different when compared to control (*p* = 0.225).

HsCRP was significantly reduced in the mangosteen group, with a mean decrease of 0.41 ± 0.34 mg/L (*p* = 0.004, −35.7 ± 22.51%). However, comparison with the control group failed to show any significant group-wise difference (Figure 3A). Similarly, fibrinogen levels had a trend decrease in the mangosteen group (−57 ± 93 mg/L, −9.9 ± 19.0%, *p* = 0.100) but failed to be significantly different when compared to control (Figure 3B).

### 3.5. Lipids Profile

HDL cholesterol levels increased significantly in the mangosteen group, suggesting an antiatherogenic effect (*p* = 0.024). However, comparison with control was not statistically significant. No change was observed regarding other serum lipids (Table 3).

### 3.6. Safety and Tolerability

In the mangosteen group, three patients over the course of the 26 weeks of follow up reported gastrointestinal (GI) symptoms, one at one month (bloating) and two at four months (diarrhea and gastric reflux respectively). In the control group, four patients experienced GI symptoms, two at one month, one at three months, and one at six months. The patients reported gastric reflux, bloating, constipation and diarrhea, respectively. None of the patients withdrew the study due to side effects. All patients recovered without treatment within a week. A cause–effect connection did not appear to exist between mangosteen treatment and GI distress.

### 3.7. Compliance

All patients in the mangosteen arm showed a good treatment compliance of at least 85% as assessed by product accountability and reported intake timing. Adherence to prescribed physical activity and diet as assessed monthly by a trained dietician did not show any significant difference between groups and did not significantly change over time (data not shown).

## 4. Discussion

We herein report that 26-week-long supplementation with mangosteen extract leads to glucose homeostasis improvement in obese insulin resistant female subjects, with a marked decrease of HOMA-IR and insulin levels, independent of BMI variations.

Mangosteen has been widely used in East Asian traditional medicine for centuries, and its favorable effects coupled with an excellent safety profile has attracted the attention of the international scientific community in recent years. In vitro and in vivo evidence has extensively proved that alpha- and gamma-mangostins are the major bioactive compounds responsible for the empirically known effects [24]. A novel possible role in the treatment of metabolic diseases has been recently suggested. Clinical trials investigating the effect of mangosteen on body weight and inflammation suggest a positive effect on both outcomes [17,18,19,20]. However, the studies are small in sample size and are of short duration (<16 weeks) and therefore need further confirmation of their reported results.

In vitro and in vivo studies prove that mangosteen also shows glucose lowering and insulin sensitizing effects [25], but no clinical trial has been conducted so far to primarily assess glycometabolic parameters. We therefore investigated the effect of mangosteen on obese insulin resistant female subjects for 26 weeks and found a striking effect on insulin resistance, whereas glucose levels at 26 weeks were not significantly changed. Of note, baseline glucose levels were normal in our population and this may have hindered the possibility of detecting a glucose lowering effect.

In vitro evidence suggests that alpha-mangostin is a potent inhibitor of pancreatic lipase and fatty acid synthase, potentially inhibiting lipids gut assimilation and fat accumulation, respectively. Previous clinical trials investigating the effect on weight loss show that mangosteen significantly reduces body weight compared to placebo [19,20]. We observed a weight reduction in the mangosteen arm that might have proved statistically significant compared to control with a wider population, longer study duration, or higher mangosteen dosage. These results suggest that mangosteen may attenuate lipogenesis or fat uptake via multiple mechanisms and may therefore prove useful to aid weight loss.

Mangosteen was also reported to reduce inflammation through several pathways, such as inhibition of conversion of arachidonic acid to prostaglandin (PG)E2 by Cyclooxygenase (COX) and inhibition of COX2 gene transcription [26,27]. Currently, the most widely used inflammatory markers are the erythrocyte sedimentation rate (ESR), an indirect method to assess plasma concentration of fibrinogen, and plasma CRP concentration. Nonetheless, ESR can be greatly influenced by the size, shape, and number of erythrocytes, as well as by other plasma constituents such as immunoglobulins. Moreover, as a patient’s condition worsens or improves, ESR changes relatively slowly and increases steadily with age, whereas CRP concentrations change rapidly and are not affected by aging. Consequently, ESR can be a misleading inflammation marker [28]. Moreover, even within the normal range, plasma CRP is an indicator for risk of chronic diseases [29]. For these reasons, we opted to monitor the inflammatory status of our patients through CRP and fibrinogen plasma levels measurement. We herein showed that hsCRP significantly decreased over time in patients taking mangosteen, unlike the control. However, group-wise comparison failed to show a significant difference between mangosteen and control groups, possibly due to the high variability of hsCRP we observed in the control group at week 26. hsCRP can in fact be greatly affected by several inflammatory conditions, and particularly in absence of an anti-inflammatory treatment, such as mangosteen, the observed effect could be more influenced by other concurring conditions, as it may have happened in our cohort. The decrease in CRP we report in the treatment arm suggests that daily consumption of mangosteen extract might be able to tame chronic inflammation of obese patients.

Currently available medications aimed at treating dyslipidemia decrease total and LDL cholesterol and triglycerides levels. Little or no effect is observed in HDL cholesterol levels [30]. The promising results we show, although failing to show a significant difference compared to control, suggest a possible role of mangosteen in selectively increasing HDL cholesterol, with its known antiatherogenic effect.

We report that mangosteen was well tolerated at the tested dosage, as there were no adverse events (clinical, laboratory, or vital sign) reasonably attributable to the product during the course of the study. This adds to the body of evidence suggesting an excellent safety profile of mangosteen.

## 5. Conclusions

In conclusion, our results suggest that mangosteen could potentially represent an appealing treatment of obesity and its comorbidities, most importantly insulin resistance, given its favorable cost/benefit ratio. However, our study has several limitations. For its pilot nature, a small number of obese insulin resistant patients who were otherwise healthy were recruited, potentially hindering the possibility of detecting significant changes, especially in regard to body weight and composition and inflammation markers. The duration of the study, although significantly longer than all other clinical trials investigating the effects of mangosteen in human subjects, is still relatively short. Also, only female subjects were enrolled, and we therefore cannot infer that the same results may be applicable to male subjects. Finally, the open label nature of the study could have led to potential bias, although it is unlikely that a placebo effect could have had any consequence on the investigated outcomes given the comparable adherence to prescribed diet and physical activity in both interventional groups.

In our opinion, the promising results we report should be further confirmed by placebo controlled wider interventional studies, possibly involving prediabetic patients and both genders, to assess whether mangosteen is not only able to improve insulin resistance in these patients but also has the ability to positively affect serum glucose levels.

## Figures and Tables

**Table 1:** Mangosteen supplement composition.

Components	Quantity (per Capsule)	Function
Mangosteen fruit pulp extract	400 mg titrated to 40% in α and γ mangostins (160 mg)	Active ingredient
Gelatin	95 mg	External coating
Magnesium salts of fatty acids and silicon dioxide	5 mg	Anti-caking

**Table 2:** General characteristics of the treatment arms. The groups were not significantly different at baseline in regard to age, BMI, body composition, glucose metabolism, and inflammatory status.

	Control (*n* = 10)	Mangosteen (*n* = 10)	*p*
Age (years)	46.00 ± 12.009	43.70 ± 2.248	0.677
BMI (kg/m^2^)	37.60 ± 7.043	37.10 ± 4.725	0.854
Body weight (kg)	101.90 ± 23.662	101.10 ± 16.690	0.931
Waist circumference (cm)	120.40 ± 15.601	115.44 ± 8.748	0.413
Body fat (%)	40.20 ± 2.781	39.60 ± 3.777	0.691
Serum glucose (mg/dL)	93.20 ± 14.250	86.20 ± 8.979	0.205
Serum insulin (mg/dL)	19.11 ± 6.431	22.40 ± 15.072	0.553
HOMA-IR	4.44 ± 1.509	4.90 ± 3.872	0.745
HbA1C (%)	5.4 ± 0.31	5.4 ± 0.23	0.876
Fibrinogen (mg/L)	360.25 ± 65.876	454.78 ± 83.215	0.071
hsCRP (mg/L)	1.00 ± 1.155	0.80 ± 0.632	0.761

**Table 3:** Lipids profile of the treatment arms at baseline and 26 weeks. HDL levels increased from baseline in the mangosteen group (*p* = 0.024), but comparison with control failed to show a statistically significant difference. No changes were observed regarding other serum lipids. C, outcome comparison of Control between 0 and 26 weeks; M, outcome comparison of mangosteen between 0 and 26 weeks; C-M, outcome comparison between control and mangosteen over time.

	Control (*n* = 10)		Mangosteen (*n* = 10)		C	M	C-M
Weeks	0	26	0	26			
	Mean ± SD	Mean ± SD	Mean ± SD	Mean ± SD	*p*	*p*	*p*
Total Cholesterol (mg/dL)	203 ± 39	199 ± 46	193 ± 28	199 ± 34	0.682	0.391	0.815
LDL-C (mg/dL)	130 ± 40	128 ± 44	121 ± 21	123 ± 31	0.796	0.724	0.779
HDL-C (mg/dL)	49 ± 14	49 ± 10	50 ± 12	58 ± 13	0.876	0.024	0.528
Triglycerides (mg/dL)	124 ± 61	111 ± 41	92 ± 30	88 ± 21	0.222	0.530	0.322
